# The F204S mutation in adrenodoxin oxidoreductase drives salinomycin resistance in *Eimeria tenella*

**DOI:** 10.1186/s13567-024-01431-6

**Published:** 2024-12-18

**Authors:** Pei Sun, Chaoyue Wang, Fujie Xie, Linlin Chen, Yuanyuan Zhang, Xinming Tang, Dandan Hu, Yang Gao, Ning Zhang, Zhenkai Hao, Yonglan Yu, Jingxia Suo, Xun Suo, Xianyong Liu

**Affiliations:** 1 National Key Laboratory of Veterinary Public Health and Safety; Key Laboratory of Animal Epidemiology and Zoonosis of Ministry of Agriculture, National Animal Protozoa Laboratory & College of Veterinary Medicine, China Agricultural University, Beijing, China; 2 Department of Pathogen Biology, Guangdong Provincial Key Laboratory of Tropical Disease Research, School of Public Health, Southern Medical University, Guangdong, China; 3 Key Laboratory of Animal Genetics, Breeding and Reproduction of the Ministry of Agriculture & Beijing Key Laboratory of Animal Genetic Improvement, China Agricultural University, Beijing, China; 4 Key Laboratory of Animal Biosafety Risk Prevention and Control (North) of MARA, Institute of Animal Sciences, Chinese Academy of Agricultural Sciences, Beijing, 100193 China; 5 College of Animal Science and Technology, Guangxi University, Nanning, 530004 China; 6 Department of Clinic Veterinary Medicine, College of Veterinary Medicine, China Agricultural University, Beijing, China

**Keywords:** *Eimeria tenella*, salinomycin, drug resistance, point mutation

## Abstract

Salinomycin is a polyether ionophore widely used for the treatment of coccidiosis in poultry. However, the emergence of coccidia strains resistant to salinomycin presents challenges for control efforts, and the mechanisms underlying this resistance in *Eimeria* remain inadequately understood. In this study, 78 stable salinomycin-resistant strains were generated through experimental evolution approaches. Whole-genome sequencing of salinomycin-resistant *Eimeria tenella* isolates revealed single nucleotide polymorphisms (SNPs), with 12 candidate genes harboring nonsynonymous mutations identified. To confirm the candidate gene responsible for conferring salinomycin resistance, we leveraged reverse genetic strategies and identified a key amino acid substitution (F204S) in adrenodoxin oxidoreductase (*EtADR*), which markedly reduced susceptibility to salinomycin. Our results elucidate the complex interactions among salinomycin resistance, parasite fitness, point mutations, and the structure of EtADR, laying the foundation for future studies on drug resistance in *Eimeria* and contributing to the development of targeted control strategies.

**Supplementary Information:**

The online version contains supplementary material available at 10.1186/s13567-024-01431-6.

## Introduction

Coccidiosis is a globally prevalent parasitic disease caused by protozoa of the genus *Eimeria* and poses a significant threat to the poultry industry and livestock [1, 2]. Current methods for the control and prevention of coccidiosis rely primarily on the use of anticoccidial drugs and vaccines [3]. Two major categories of anticoccidial drugs, polyether antibiotics and synthetic compounds, are widely employed in the poultry industry [4]. However, the indiscriminate and excessive use of these drugs has led to the emergence of drug-resistant *Eimeria* strains, rendering certain drugs ineffective [5–8]. The presence of drug-resistant strains complicates disease control efforts, thereby limiting available options for the prevention and treatment of coccidiosis. This highlights the urgent need for the development of novel anticoccidial drugs with unique mechanisms of action to combat drug-resistant *Eimeria*.

A comprehensive understanding of the molecular markers and underlying mechanisms of drug resistance in *Eimeria* is crucial for the development of effective control measures. Our previous research successfully demonstrated that mutated prolyl-tRNA synthetase (*PRS*) confers resistance to halofuginone in *Eimeria* [9]. Additionally, there is evidence linking mutations in the cytochrome b gene to resistance against decoquinate in *Eimeria* [10]. Although only a limited number of molecular markers have been identified in coccidia, extensive research on other Apicomplexan parasites, such as *Plasmodium falciparum* and *Toxoplasma gondii*, has successfully elucidated multiple molecular markers and resistance mechanisms [11–17]. Insights from these studies suggest that combining directed evolution with whole-genome sequencing could be a powerful strategy to identify targets and resistance pathways responsible for specific phenotypes in *Eimeria*. Recently, reverse genetic approaches, such as genome-scale CRISPR screens, have been developed for parasites to discover candidate mutations [18]. These strategies hold great potential for identifying new targets for anticoccidial drug development and for tracking the emergence and spread of drug-resistant strains in the field.

Salinomycin, a monocarboxylic polyether antibiotic derived from *Streptomyces albus*, functions by catalyzing the exchange of Na^+^ for K^+^ across biological membranes [19]. For decades, ionophores have been the primary option for controlling coccidiosis due to their slow resistance development. Salinomycin is considered the least toxic ionophore and exhibits broad-spectrum anticoccidial activity against all *Eimeria* species [1]. Previous studies have demonstrated that salinomycin-resistant *Eimeria* strains are prevalent in various regions, including the USA and Korea. Additionally, multiple *Eimeria* species have been reported to exhibit resistance to salinomycin [1, 20, 21]**.** The emergence of salinomycin-resistant strains in various regions has significantly reduced drug effectiveness [20–28]. Some studies have suggested a potential association between salinomycin resistance and ABC transporters, a group of membrane proteins involved in the efflux of various substances [29]. Unfortunately, recent studies on anticoccidial drug-resistant *Eimeria* have focused primarily on transcriptomic and epidemiologic investigations [27, 28, 30–33].

Despite the challenges that exist in studies of resistance mechanisms in *Eimeria*, here, we elucidated a point mutation in adrenodoxin oxidoreductase (*EtADR*) that reduces the susceptibility of *Eimeria tenella* to salinomycin. These findings shed light on the molecular changes associated with salinomycin resistance in *E. tenella* and represent a significant step toward understanding the detailed resistance mechanism of this important drug. Moreover, our research offers potential avenues for developing effective strategies to combat drug resistance in *Eimeria* infections.

## Materials and methods

### Animals and parasites

Acre Arbor broilers were purchased from Beijing Arbor Acres Poultry Breeding (Beijing, China), and chickens were used for proliferation, drug-resistant strain selection and candidate gene verification. All the birds were given a drug-free diet and water ad libitum unless the experiment was performed. *E. tenella* Houghton was maintained in the laboratory, and the procedures for oocyst collection, purification and sporulation were carried out as described in previous work [34]. Cervical dislocation, which aims to cause rapid loss of consciousness in chickens, was performed for the chickens necessary for sacrifice.

### Selection and characterization of salinomycin-resistant strains

Two different approaches were used in our experiment to induce salinomycin-resistant strains. To obtain resistant strains rapidly, 300 1 day-old chickens were equally divided into two groups. The chickens in group 1 were inoculated with 500 sporulated wild-type *E. tenella* Houghton, while the chickens in group 2 were fed 60 mg/kg salinomycin (working concentration) during the experiment. The detailed procedures were performed as previously described [9].

To obtain intermediate strains during induction, the wild-type strain was induced by gradually increasing the concentration of salinomycin from 20 mg/kg to 240 mg/kg over 20 passages, and the resistant strain was completely resistant to 240 mg/kg (fourfold) salinomycin. After three generations of relaxed selection, the resistant strains were tested for the resistant phenotype. Comparative studies on the reproducibility of salinomycin-resistant and salinomycin-sensitive strains incorporating modifications based on previous research have been conducted [9].

### Genome sequence sample preparation and analysis

Parasites of the wild-type, intermediate, and salinomycin-resistant strains were extracted via cetyltrimethylammonium bromide (CTAB) as previously reported [35]. The resulting whole-genome sequencing libraries were normalized and sequenced via the Illumina HiSeq-3000 platform as paired-end reads extending 150 bases from both ends of the fragments. Clean data were aligned to the *E. tenella* Houghton reference genome (pEimTen1.1) via BWA mem under the default parameters. The Genome Analysis Toolkit GATK v4 was used to call SNPs and combine vcfs with the default parameters [36]. The R package QTLseqr was subsequently used for ΔSNP index data visualization. The detailed methods were described previously [37]. The WGS data are available in the NCBI SRA database under accession number PRJNA1012853.

### Plasmid construction

*His-candidate gene-EYFP-Actin* All the overexpression plasmids used in this study were derived from HCYA (eCas9-NLS-2A-YFP), and the Cas9 position was replaced with different candidate genes. If the length of the candidate genes exceeded 2000 bp, P2A was inserted between the candidate gene and EYFP. For candidate genes shorter than 2000 bp, Flag-Linker was used to connect the candidate genes and EYFP.

*U6-sgRNA-5HR-mCherry-3HR* To generate a homologous recombinant plasmid, a gRNA was designed to target the N-terminal region of the candidate gene (ToxoDB: ETH2_0637800), and the gRNA was cut within a 50-bp window upstream of the start codon. The plasmid also harbored the fluorescent protein mCherry, which was integrated into the 5’ end of the target gene. The strategy was designed as previously described [38]. All primers used are provided in Additional file 7.

All the fragments and the T-vector in all the plasmids were linked via a seamless assembly strategy (pEASY^®^-Uni Seamless Cloning and Assembly Kit).

### Parasite transfection

The transfection procedures were performed with the SnaBI restriction enzyme as described in a previous report [9, 39–41]. To generate overexpressing parasites, parasites were transfected with different overexpression plasmids and then mixed. Mixed parasites were subsequently inoculated into 15 2 week-old chickens, and the wild-type group and mutation group were fed separately. Throughout the entire experiment, the chickens were fed 120 mg/kg salinomycin. To generate homologous recombinant strains, the stable eCas9-expressing *E. tenella* line was transfected with the homologous recombinant plasmid.

To enrich the positive transgenic parasites, positive sporocysts were collected through flow cytometry and then inoculated with new chickens under salinomycin (120 mg/kg) selection.

### Indirect immunofluorescence assay (IFA)

IFA was performed to characterize the location and expression of the candidate gene via the following protocol. The transgenic sporozoites were extracted and purified through a cellulose filter and then infected with HFF cells (human foreskin fibroblasts) for approximately 4 h, after which PBS was used to wash out uninvaded sporozoites. Intracellular sporozoites were fixed with 4% paraformaldehyde (PFA), permeabilized with 0.25% Triton-100 for 15 min, and then incubated with 3% BSA for 15 min at 37 ℃. To verify the overexpression of parasites and homologous recombinant parasites, GFP-tag rabbit polyab (1:200), anti-mCherry (ab183628, 1:100), Cy3-conjugated goat anti-mouse IgG (1:200), FITC-conjugated goat anti-rabbit IgG (1:200) and Hoechst 33258 (1:100) antibodies were used. The monolayers were observed with a Leica confocal microscope (Leica, YCS SP52, Germany) at 633 × magnification, and high-content imaging and analyses were performed with LAS AF lite 2.2.0 software.

### Western blot

For western blotting, total protein was extracted from the parasites via a standard procedure. GFP-tag rabbit polyab (1:500), anti-mCherry antibody (1:1000), horseradish peroxidase (HRP)-conjugated goat anti-mouse IgG (1:2000) and HRP-conjugated goat anti-rabbit IgG (1:2000) were used to detect the expression level of the candidate gene. The actin of *E. tenella* was used as a control.

### Phylogenetic analysis of ADR

Protein sequences containing the pyr_redox domain were obtained for representative apicomplexan genomes from NCBI on the basis of their annotation with the SMART domain. The homologous proteins were aligned via ClustalW, and the phylogenetic tree was generated via the neighbour‒joining method [42]. Bootstrap values were calculated for 10 000 trials. Visualizations were generated via Chiplot.

### Molecular docking

All structural superposition and preparation of figures were performed via PyMOL. The 3D models of the proteins were predicted via AlphaFold2, and the Ramachandran plot was analysed via PyMOL. To characterize the active pockets, the 3D structure of ADR was prepared by adding bond orders, hybridization, explicit hydrogens, charges, and Tripos atom types. The molecular docking data were analysed by AutoDock, and poses showing a similar conformation (root mean square deviation [RMSD] of < 2.0 Å), as assessed by AutoDock, were retained, and the first-ranking pose was chosen for further analysis.

### Statistical analysis

GraphPad Prism 8.0 was used to generate graphs and analyse the statistical data. All the data were analysed with two-tailed Student’s *t* tests. *p* < 0.05 was considered to indicate statistical significance.

## Results

### Obtention of salinomycin-resistant strains of *E. tenella* by experimental evolution

**Figure 1:**
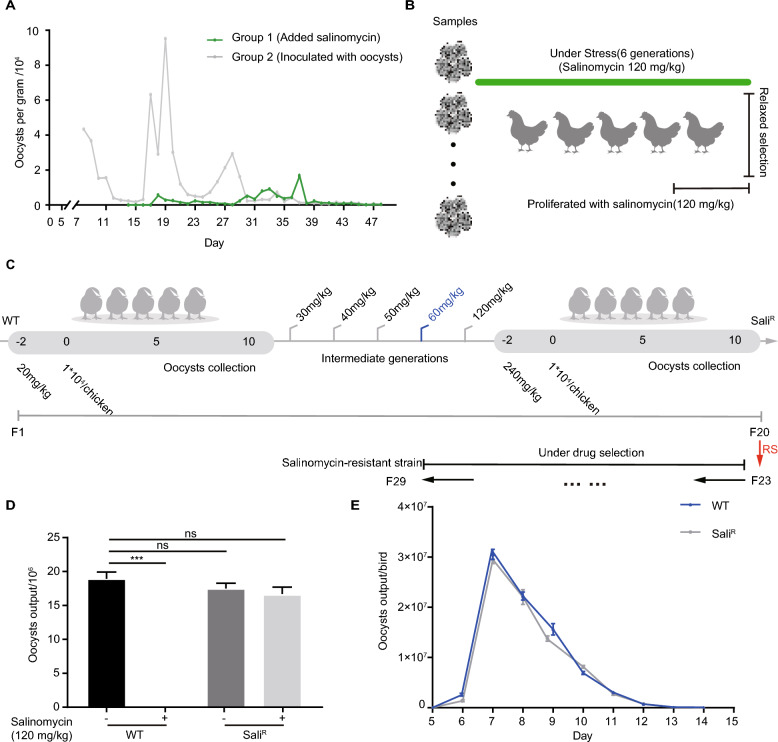
**Selection and characterization of salinomycin-resistant** ***E. tenella* strains.** **A** Oocyst output curves of chickens in the initially infected group (group 1, gray line) or salinomycin-treated group (group 2, green line). **B** Workflow used to obtain stable salinomycin-resistant strains. **C** Selection of salinomycin-resistant strains via a dose-escalation strategy. RS, relaxed selection. **D** Comparison of the oocyst output between groups of chickens inoculated with salinomycin-resistant and -sensitive strains and fed with/without salinomycin (120 mg/kg). Four groups of chickens were set during the entire experiment. Each bird was inoculated orally with 1 × 10^4^ oocysts of either the sensitive or resistant strain. Each group had three replicates. ***p < 0.001. **E** Oocyst output curves of birds infected with salinomycin-resistant or salinomycin-sensitive strains. Each bird was inoculated with 500 fresh sporulated oocysts, and the resistant group was fed 120 mg/kg salinomycin throughout the entire experiment, while no drug was inoculated into the sensitive group. Each experiment was performed in triplicate ***p < 0.001.

To obtain salinomycin-resistant strains, we initially employed an accelerated experimental evolution method to select resistant strains [9]. Consistent with previous findings, the peak oocyst output peak in the group treated with salinomycin occurred at approximately 37 dpi (Figure 1A, Additional file 1). To amplify the population of salinomycin-resistant strains, the ^second^ experiment was conducted, resulting in the collection of 80 samples. To stabilize the resistant phenotype, the oocysts were propagated at a concentration of 120 mg/kg, subjected to three passages under relaxed selection, and then exposed to a high concentration of salinomycin (Figure 1B). Additionally, to monitor the dynamic emergence of the salinomycin-resistant phenotype, a dose-escalation process was implemented, leading to the acquisition of resistant strains (Figure 1C, Additional file 2). After selection, a total of 78 strains were collected.

To evaluate the reproductive capacity of salinomycin-resistant strains, we compared the oocyst output of resistant and sensitive strains, both in the presence and absence of salinomycin (120 mg/kg). The results revealed no significant difference in oocyst production between the salinomycin-treated resistant strain and the untreated sensitive strain (Figure 1D). In contrast, no oocysts were detected in the sensitive group under drug pressure (Figure 1D). Moreover, we generated oocyst output curves to compare the endogenous development of different strains, and the results were consistent with the aforementioned data (Figure 1E). Overall, we successfully utilized two experimental evolution strategies to obtain 78 stable salinomycin-resistant strains.

### Identification of candidate genes associated with salinomycin resistance in *E. tenella* by resequencing and SNP analysis

Research has demonstrated that pathogens are exposed to a range of drug concentrations, which creates varying selective pressures. When drug concentrations are sufficiently high to inhibit pathogen growth, the presence of preexisting resistant mutants becomes crucial. The rate at which these resistant mutants become enriched is influenced by factors such as their prevalence within the population and their fitness levels [43].

**Figure 2:**
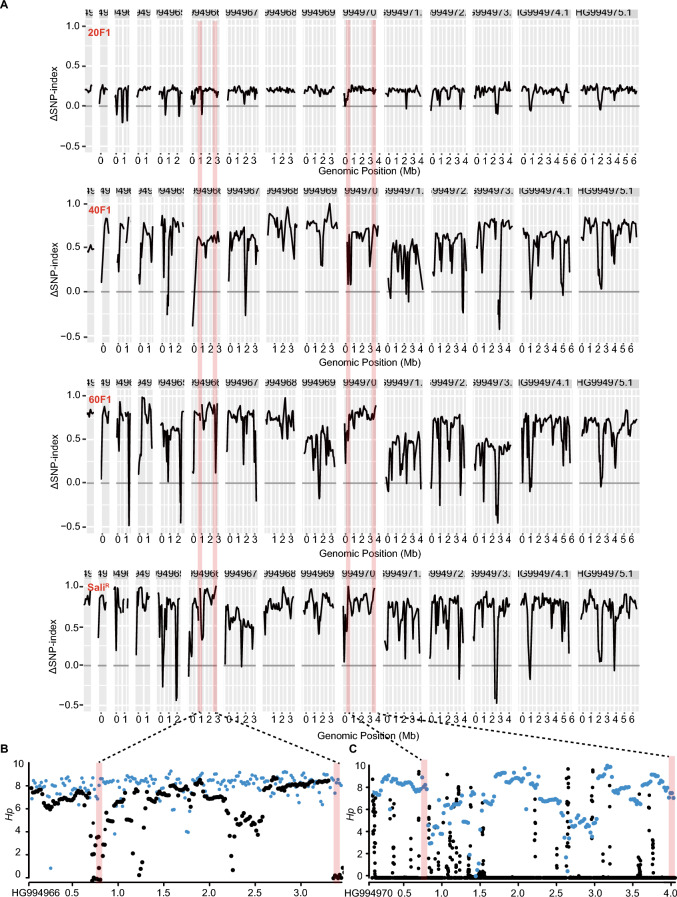
**Whole-genome sequencing of selective sweeps among salinomycin-resistant and -sensitive strains.** **A** *ΔSNP indices* for intermediate- and resistant strains identified via QTL-seq. Plots produced by the plotQTLStats () function with a 1 Mb sliding window. Red line, candidate loci. **B** A sliding window of average *Hp* on chromosomes HG994966 and HG994970. A 20 kb sliding window with a 10 kb step is depicted. The average *Hp* of the intermediate generation is blue, while that of the salinomycin-resistant strain is also shown as black dots for comparison. Red line, candidate loci.

Whole-genome sequencing was used to investigate the candidate mutations among different generations. Following a selective sweep across the entire genome, beneficial mutations become fixed, leading to a reduction in genetic diversity around the selected locus [44]. The *ΔSNP index* was used to analyse the allele frequency in the resistant and intermediate generations across the genome via a 50 kb sliding window, with loci exhibiting high allele frequencies among intermediate generations excluded (Additional file 3 and Additional file 4). Additionally, heterozygosity (*Hp*) data were used to assess the diversity of candidate loci. Only candidate loci with a mutation frequency > 0.95 and *Hp* < 0.5 were considered, and intergenic regions were excluded. Through this stringent filtering process, only four candidate loci on two chromosomes (HG994966 and HG994970) were identified (Figures 2A–C). From these candidate regions, nonsynonymous mutations were further analysed, leading to the identification of 12 candidate genes (Table 1). The consistent results obtained from both the *ΔSNP index* and *Hp* data suggest that one or more of these 12 genes may be associated with salinomycin resistance.

**Table 1:** **Whole-genome sequencing identified point mutations in 12 candidate genes**

Chromosome	Gene ID	Annotation	No. of SNPs/nonsynonymous	Mutations	Amino acid change
HG994966	ETH2_0636700	ABC transporter	11/5	A3073G, A3062C, C3050T, C2993T, G2343C	T1025, G102A, T1017, T998I, M781
	ETH2_0637000	Aldehyde dehydrogenase family	4/1	G1108C	V1370L
	ETH2_0637500	Hypothetical protein	7/2	A1138G, A1119T	I380V, L373P
	ETH2_0637600	Hypothetical protein	1/1	G991A	G331L
	ETH2_0637800	Adrenodoxin oxidoreductase	1/1	T611C	F204S
	ETH2_0638400	Hypothetical protein	6/2	G1848T, A602G	M616I, A201S
	ETH2_0638700	Hypothetical protein	2/2	A86G, G56A	A29S, S18A
HG994970	ETH2_1005600	Hypothetical protein	6/1	G1912A	V163M
	ETH2_1004500	Hypothetical protein	7/2	A2711G, G2671A	L904R, V891I
	ETH2_1045600	Hypothetical protein	7/5	C1742T, T1696C, A1694T, C1683G, T1670C	S581L, S566P, H565L, H561G, I557T
	ETH2_1004800	ABC transporter	–	Deletion	–
	ETH2_1045800	Hypothetical protein	6/2	G694A, T698C	A232T, V233A

### Validation of candidate genes conferring resistance to salinomycin in *E. tenella* by pool transfection

**Figure 3:**
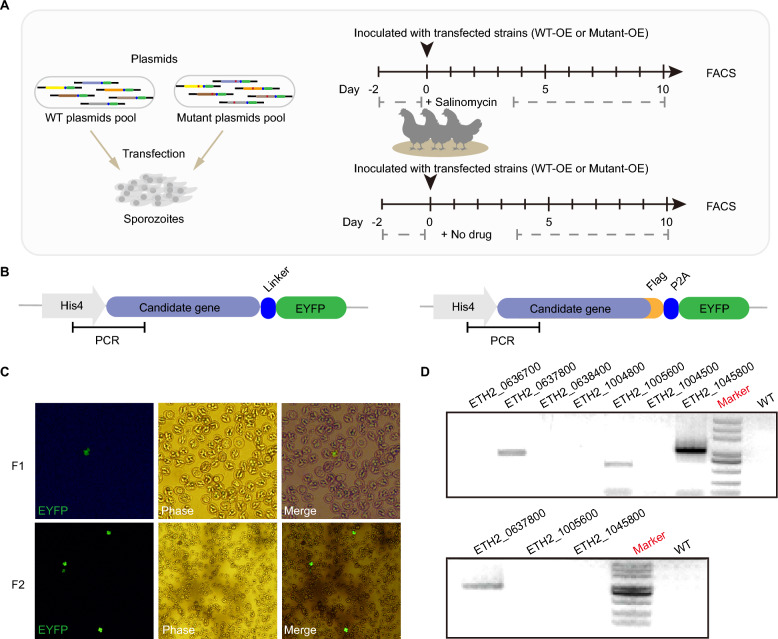
**Verification of candidate genes associated with salinomycin resistance via a pool-transgenic approach.** **A** Schematic illustration of the pool-based transgenic strategy. **B** Schematic illustration of the construction of pool-overexpressing strains. The PCR method involves specific primers designed for the validation of transfected strains. **C** Fluorescence observation of 1^st^ and 2^nd^ generations of transgenic oocysts under a confocal microscope. Scale bar, 5 μm. **D** Validation of the transgenic strains via PCR. Marker, AL5000.

To validate the potential role of candidate genes in conferring salinomycin resistance, we employed a pool-transfection approach to assess the involvement of specific salinomycin resistance target genes among these 12 candidates (Figure 3A). Overexpression plasmids containing either the mutated or the wild-type (sensitive) versions of these genes were constructed (Figure 3B). To monitor the growth of the transgenic parasites, we used a combination of salinomycin selection and fluorescence markers to determine whether the mutations in the candidate genes conferred resistance to the drug (Additional file 5).

As expected, transgenic parasites harbouring the mutant gene exhibited fluorescence after salinomycin selection, and the fluorescence was significantly enriched in the second generation (Figure 3C). Specific primers were designed for each plasmid, and genomic DNA was extracted from the 1^st^ and 2^nd^ progeny to confirm the presence of the target gene(s) (Figure 3D). On the basis of these data, the ETH2_0637800 gene, annotated as adrenodoxin oxidoreductase (*EtADR*), harboring a single mutation site, T611C, resulting in an amino acid substitution, F204S, may confer resistance to salinomycin in *E. tenella*. Therefore, we speculated that mutated *EtADR* may lead to salinomycin resistance in *Eimeria*.

### Single-point mutation (T611C) in *EtADR *decreases salinomycin susceptibility in *E. tenella*

**Figure 4:**
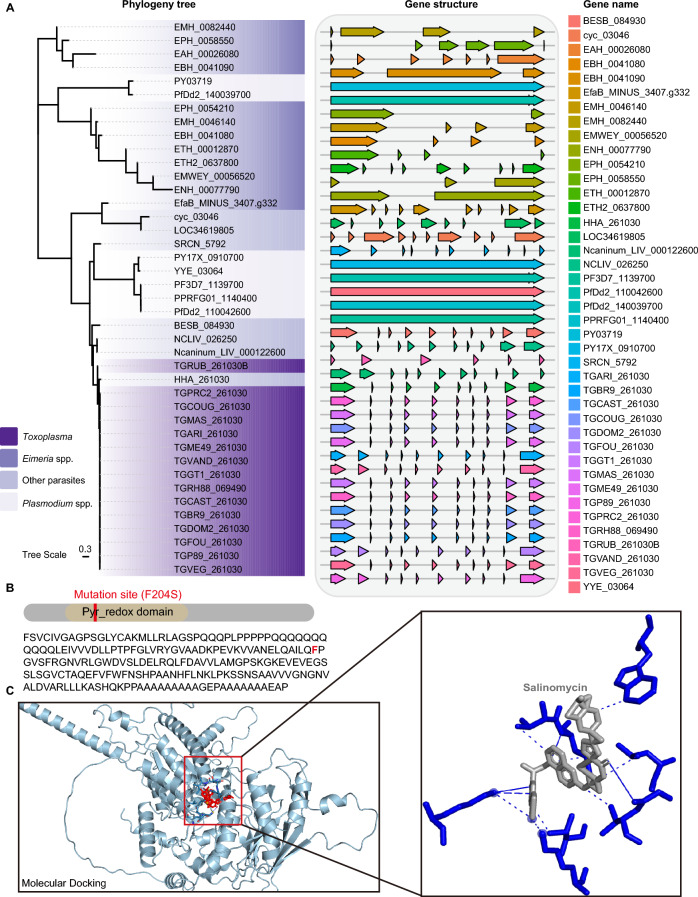
**Phylogenetic tree and molecular docking of the candidate protein ADR.** **A** Phylogenetic tree of ADR proteins from different species. The neighbor-joining phylogenetic tree was constructed via MEGA 11. The alignment was performed via ClustalW. The scale bar represents substitutions per site. **B** Gene model of the *EtADR* gene in *E. tenella*. Brown modules represent the Pyr_redox domain. Red line, mutation site. **C** Crystal structure visualization of the combination of the ADR^Mut^ protein and the drug salinomycin. The diagram on the right shows the active pocket.

These results suggest a potential relationship between *EtADR* and salinomycin resistance. To further investigate whether the T611C mutation in *EtADR* decreases susceptibility to salinomycin in *E. tenella*, we conducted a series of analyses. Multiple sequence alignments revealed that this protein is conserved among apicomplexan parasites and contains a single domain, pyr_redox, which is an NADH-binding domain primarily responsible for the oxidation‒reduction process of the protein [45] (Figures 4A and B). We then performed molecular docking analysis to examine whether the point mutation introduced by the T611C mutation affected the docking form of EtADR (Additional file 6). Interestingly, the mutated residue was located in the “active pocket” of the protein, suggesting that this mutation could directly influence the binding and activity of EtADR (Figure 4C). On the basis of these results, we speculate that the T611C mutation in EtADR may contribute to salinomycin resistance in *E. tenella.*

### ***EtADR***^Mut^ confers salinomycin resistance in ***E. tenella,*** as validated through overexpression and homologous recombination strategies

**Figure 5:**
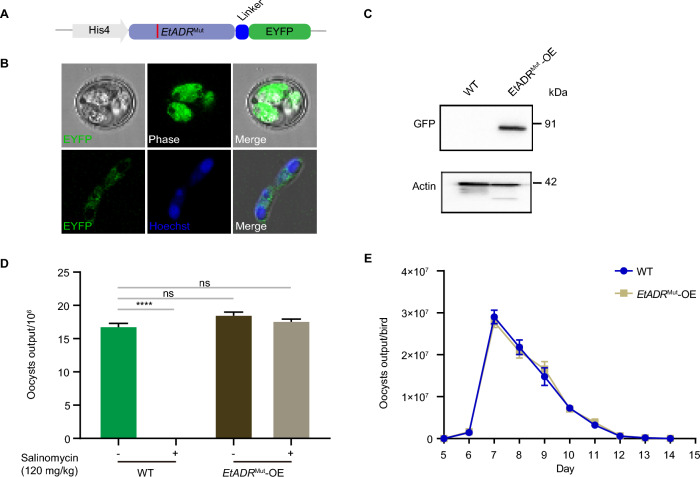
**OE strategies for verifying that EtADR**^**Mut**^** is related to salinomycin resistance in** ***E. tenella*.** **A** Schematic illustration of constructing the EtADRMut-OE strain. Red line, mutation site. **B** Fluorescence observation of *EtADR*^Mut^-OE transgenic oocysts under a confocal microscope. IFA shows the localization of *EtADR*^Mut^. Scale bar, 5 μm. **C** Western blot of total protein extracted from the parental and *EtADR*^Mut^-OE strains. PVDF membranes were probed with anti-GFP to detect the presence of *EtADR*^Mut^ protein (upper panel), and anti-EtActin was used as a control for normalization (lower panel). **D** Comparison of oocyst output between wild-type and transgenic strains with/without salinomycin (120 mg/kg). Each group had three replicates. ***p < 0.001. **E** Oocyst output curves of the wild-type and transgenic strains. Each experiment was performed in triplicate. ***p < 0.001.

To further establish the functional link between the point mutation and the resistant phenotype, we introduced the point mutation into the wild-type strains. First, we constructed an overexpression transgenic strain harboring *EtADR*^Mut^ (Figure 5A). We employed a combination of drug selection and fluorescence-based screening methods to select positive transgenic strains that became stable after five successive generations under selective pressure. To validate the expression and subcellular localization of the ADR protein, we performed IFA and Western blot analysis (Figures 5B and C). We then conducted a comparative analysis to assess the reproductive capacity of the transgenic strains in comparison with that of the wild-type strains in the presence or absence of salinomycin (120 mg/kg). Our results revealed no significant difference in reproductive capacity between the overexpression strain treated with salinomycin and the wild-type strain without drug exposure (Figure 5D). Consistent with these findings, the oocyst output curve of the transgenic strains treated with salinomycin closely resembled that of the wild-type strain without drug exposure (Figure 5E).

**Figure 6:**
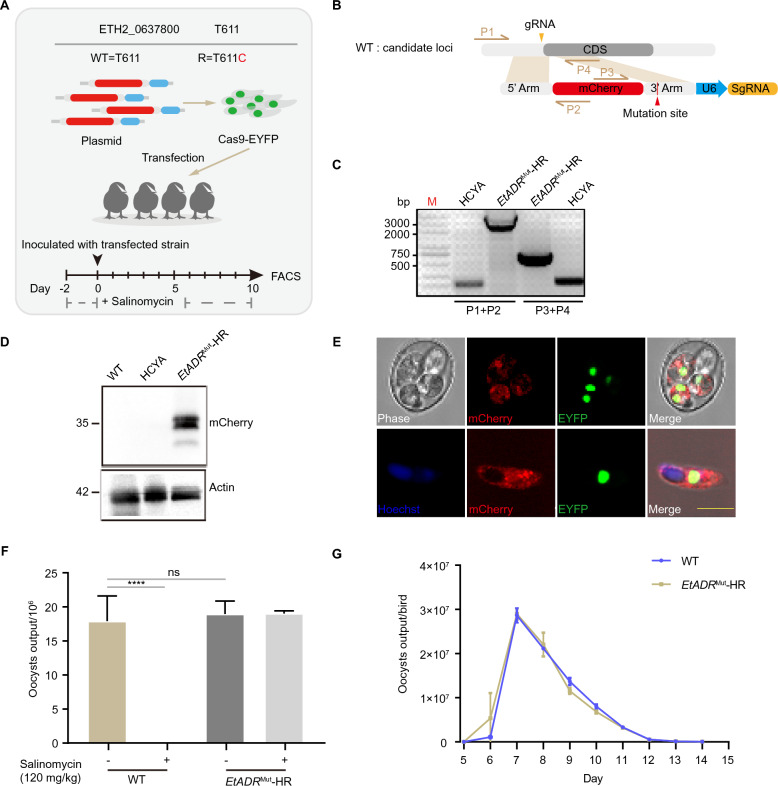
**Construction of a homologous recombination (HR) strain via CRISPR/Cas9.** **A**–**C** Schematic illustration of the CRISPR/Cas9 genome-editing strategy used to introduce a point mutation into the wild-type *E. tenella* chromosome for the *EtADR* gene. The wild-type allele is T611, whereas the mutant is 611C. Double-positive parasites were isolated via FACS and then propagated under salinomycin selection. The edited loci in the genomes of the progeny oocysts were confirmed via PCR. **D** Western blot of total protein extracted from the parental and *EtADR*^Mut^-HR strains. **E** Fluorescence observation of transgenic oocysts under a microscope. IFA shows the localization of *EtADR*^Mut^. Scale bar, 5 μm. **F** Comparison of the total oocyst output between the wild-type and *EtADR*^Mut^-HR strains with/without salinomycin (120 mg/kg). **G** Oocyst output curves of the wild-type and *EtADR*^Mut^-HR strains.

Additionally, we employed another genome-editing approach based on CRISPR/Cas9 technology to introduce nonsynonymous SNPs into the native loci of the wild-type parental line (Figures 6A and B). Specific primers were designed to verify homologous recombination (Figure 6C). The EtADR^Mut^ protein, identified by tagging, was detected only in the transgenic strains via immunofluorescence or immunoblotting, indicating that we successfully integrated the mutant gene into the genome of the wild-type parasite strain (Figures 6D and E). To investigate the phenotype of these transgenic strains, we compared the oocyst output and endogenous development between the parental and transgenic strains. The results demonstrated that the transgenic strains remained stable even under drug pressure (Figures 6F and G). These findings provide compelling evidence that the introduction of single-point mutations in the original resistant populations can significantly decrease the susceptibility of *E.* *tenella* to salinomycin.

## Discussion

Currently, drug resistance continues to emerge, diversify, and spread *in Eimeria* [2]. Although the impact of anticoccidial drugs on epidemiology has been largely studied, interest in the molecular mechanisms of drug resistance has only recently gained traction within the scientific community [9, 22, 31–33]. Notably, other apicomplexan parasites, *Plasmodium* and *Toxoplasma*, exhibit molecular markers of different drug resistance [11, 12, 18, 43]. However, only a few molecular markers have been described in *Eimeria*, such as the mutated *EtcPRS* and cytochrome b [9, 10]. In our study, we employed a combination of forward and reverse genetic approaches to pinpoint that *EtADR*^Mut^ is correlated with salinomycin resistance in *Eimeria*. Together, our research provides new insights into the molecular mechanisms underlying the development of salinomycin resistance.

Previous studies examining SNPs across different strains of *Toxoplasma* and *Plasmodium*—which were passaged an unknown number of times in various laboratories—have suggested that minimizing irrelevant mutation site interference requires either increasing the number of samples or tracking dynamic changes by gradually increasing drug concentrations to derive parasites with elevated drug tolerance [12, 38]. Research conducted on the model organism *Escherichia coli* has demonstrated that strong selective pressures, even under homogeneous conditions, lead to spontaneous mutations that often confer a fitness advantage and eventually become fixed in the population [46]. Our study employed two experimental evolution strategies to screen for drug-resistant strains. The traditional gradient induction strategy helps clarify the dynamic changes in the mutation frequency of resistance loci during the induction process, whereas the rapid induction strategy facilitates the quick selection of multiple resistant strains in a short period. Genomic analysis of these resistant strains has allowed for the rapid elimination of confounding loci [9, 11, 12]. On the basis of these data, only 12 candidate genes were screened.

In *Eimeria*, traditional transfection techniques suffer from in vivo screening and low-efficiency transfection challenges. To address these limitations, we developed a novel transgenic strategy using pool-transfection with 12 mutated-overexpression plasmids. This approach significantly reduced the number of chickens required and saved time. To further investigate the importance of point mutations in *EtADR*, we introduced this mutation into a wild-type background via CRISPR/Cas9 [47, 48]. This outcome is similar to the phenotype observed in the *EtADR*^Mut^ overexpression strain, in which “resistant” lines presented significantly decreased susceptibility to salinomycin. While traditional forward and reverse approaches can be time-consuming, they may fail to detect minor mutations or those that negatively impact parasite fitness and can be used only for positive selection schemes. Recently, whole-genome CRISPR screening technology has provided new opportunities for studying different phenotypes across various species. This technology could be applied to emerging model organisms with interesting biological properties or biotechnological applications, ranging from mammals to microorganisms [49]. However, in *Eimeria*, low transfection efficiency and in vivo screening pose challenges for CRISPR screening. Therefore, improving the technology for CRISPR screening in *Eimeria* would offer a versatile approach for large-scale functional analysis, bridging the gap between phenotypes and candidate genes. Additionally, enhancing CRISPR-based screens in *Eimeria* could help uncover drug resistance mechanisms and aid in the design of new therapeutic targets.

In summary, our study paves the way for further exploration of drug resistance in *Eimeria* and identifies a novel molecular marker for anticoccidial drugs. The discovery of this marker facilitates the rapid identification of resistant strains in the field and supports the development of new anticoccidial drugs targeting this site, ultimately improving the effectiveness of clinical prevention and control strategies. These findings provide a solid foundation for future research on drug resistance in *Eimeria* and contribute to the formulation of strategies to address this escalating challenge.

## Supplementary Information

**Additional file 1. Evaluation of parasite resistance in the salinomycin group. **To determine whether the oocysts at the peak timepoint in the salinomycin-treated group presented drug resistance phenotypes, the oocysts were inoculated into three groups of chickens. These chickens were subjected to salinomycin pressure for proliferation. The number of oocysts produced by each group was counted and compared with that of the sensitive group.

**Additional file 2. Acquirement of salinomycin-resistant strains under dose escalation. **To obtain intermediate strains during induction, the wild-type strain was induced by gradually increasing the concentration of salinomycin from 20 mg/kg to 240 mg/kg over 20 passages, and the resistant strain was completely resistant to 240 mg/kg (4-fold) salinomycin.

**Additional file 3. ΔSNP index analysis of intermediate generations of parasites in the process of experimental evolution. **(A-E) QTL analysis of intermediate-generation parasites induced by salinomycin. Quantitative trait loci (QTLs) for salinomycin resistance identified via QTLseq. Plots produced by the plotQTLStats() function with a 20-kb sliding window.

**Additional file 4. ΔSNP index analysis of Salinomycin-resistant strains obtained through experimental evolution. **(A-D) QTL analysis of strains resistant to salinomycin. Plots produced by the plotQTLStats() function with a 20-kb sliding window.

**Additional file 5. Acquisition of positive transgenic strains under drug and fluorescence selection. **To determine the percentage of positive transgenic parasites, positive sporocysts were collected through flow cytometry and then inoculated with new chickens under salinomycin (240 mg/kg) selection.

**Additional file 6. 3D structure evaluation and basic character prediction. **(A) THMHH results for the EtADR protein. Analysis was conducted using the entire amino acid sequence of the *EtADR* gene. Plots for TMHMM are presented as the probability (y-axis) of an amino acid (x-axis) residue sitting in the helix, inside, or outside summed over all possible model paths. (B) Hydrophobicity plot of the ADR protein. The hydrophobicity values were determined via the method of Kyte and Doolittle. (C) Ramachandran plot analysis of the EtADR 3D protein. The plot calculations were computed via the PROCHECK server. The red regions in the graph indicate the most allowed regions [A, B, L], additional allowed regions [a, b, l, p] are indicated in brown, and generously allowed regions [~a, ~b, ~l, ~p] are indicated in yellow. (D) Details of the residue number in each region of the Ramachandran plot and the G‐score.

**Additional file 7. Primers used in this study.**

## Data Availability

All the data generated or analysed during this study are included in this published article.
