# Supplementary material for: The F204S mutation in adrenodoxin oxidoreductase drives salinomycin resistance in Eimeria tenella
Source: Vet Res. 2024 Dec 18;55:170. doi: 10.1186/s13567-024-01431-6 (PMC11654014; doi:10.1186/s13567-024-01431-6)
Supplement: Supplementary file 1 — Additional file 1. Evaluation of parasite resistance in the salinomycin group. To determine whether the oocysts at the peak timepoint in the salinomycin-treated group presented drug resistance phenotypes, the oocysts were inoculated into three groups of chickens. These chickens were subjected to salinomycin pressure for proliferation. The number of oocysts produced by each group was counted and compared with that of the sensitive group. [file 13567_2024_1431_MOESM1_ESM.docx]

**Additional file 1. Evaluation of the drug resistance of parasites in the salinomycin group.**

| **Samples** | **No. of chicken** | **Week (w)** | **Dose of inoculation** | **Drug concentration** | **Oocysts output** |
| --- | --- | --- | --- | --- | --- |
| Oocysts collected at 35 dpi | 3 | 4 | 1.5 × 10^4^ | 60 mg/kg | 4.13 × 10^6^ |
|  | 3 | 4 | 1.5 × 10^4^ | 60 mg/kg | 6.21 × 10^6^ |
|  | 3 | 4 | 1.5 × 10^4^ | 60 mg/kg | 4.98 × 10^6^ |
|  | 3 | 4 | 1.5 × 10^4^ | - | 8.9 × 10^7^ |
| WT | 3 | 21 | 1.5 × 10^4^ | 60 mg/kg | - |
