# Supplementary material for: The F204S mutation in adrenodoxin oxidoreductase drives salinomycin resistance in Eimeria tenella
Source: Vet Res. 2024 Dec 18;55:170. doi: 10.1186/s13567-024-01431-6 (PMC11654014; doi:10.1186/s13567-024-01431-6)
Supplement: Supplementary file 2 — Additional file 2. Acquirement of salinomycin-resistant strains under dose escalation. To obtain intermediate strains during induction, the wild-type strain was induced by gradually increasing the concentration of salinomycin from 20 mg/kg to 240 mg/kg over 20 passages, and the resistant strain was completely resistant to 240 mg/kg (4-fold) salinomycin. [file 13567_2024_1431_MOESM2_ESM.docx]

**Additional file 2. Acquirement of salinomycin-resistant strains under dose-escalation.**

| **Drug concentration (mg/kg)** | **Generation** | **Oocysts output**  **(Total)** | **Oocysts output**  **(Acquire)** |
| --- | --- | --- | --- |
| 20 | F1 | 2.32 × 10^6^ | 1.887 × 10^6^ |
|  | F2 | 4.17 × 10^7^ | 3.52 × 10^7^ |
|  | F3 | 6.44 × 10^7^ | 5.09 × 10^7^ |
| 30 | F4 | 4.62 × 10^5^ | 3.79 × 10^5^ |
|  | F5 | 2.31 × 10^7^ | 1.85 × 10^7^ |
|  | F6 | 5.51 × 10^7^ | 4.35 × 10^7^ |
| 40 | F7 | 1.43 × 10^6^ | 1.05 × 10^6^ |
|  | F8 | 5.32 × 10^7^ | 4.05 × 10^7^ |
|  | F9 | 8.18 × 10^7^ | 6.3 × 10^7^ |
| 50 | F10 | 2.43 × 10^5^ | 1.75 × 10^5^ |
|  | F11 | 4.18 × 10^7^ | 3.35 × 10^7^ |
|  | F12 | 9.36 × 10^7^ | 7.35 × 10^7^ |
| 60 | F13 | 3.14 × 10^6^ | 1.98 × 10^6^ |
|  | F14 | 6.17 × 10^7^ | 4.51 × 10^7^ |
|  | F15 | 8.91 × 10^7^ | 7.06 × 10^7^ |
| 120 | F16 | 2.3 × 10^7^ | 1.41 × 10^7^ |
|  | F17 | 6.61 × 10^7^ | 3.74 × 10^7^ |
|  | F18 | 7.09 × 10^7^ | 5.89 × 10^7^ |
| 240 | F19 | 4.11 × 10^7^ | 3.25 × 10^7^ |
|  | F20 | 8.3 × 10^7^ | 6.89 × 10^7^ |
