# Supplementary figures and images for: The F204S mutation in adrenodoxin oxidoreductase drives salinomycin resistance in Eimeria tenella
Source: Vet Res. 2024 Dec 18;55:170. doi: 10.1186/s13567-024-01431-6 (PMC11654014; doi:10.1186/s13567-024-01431-6)

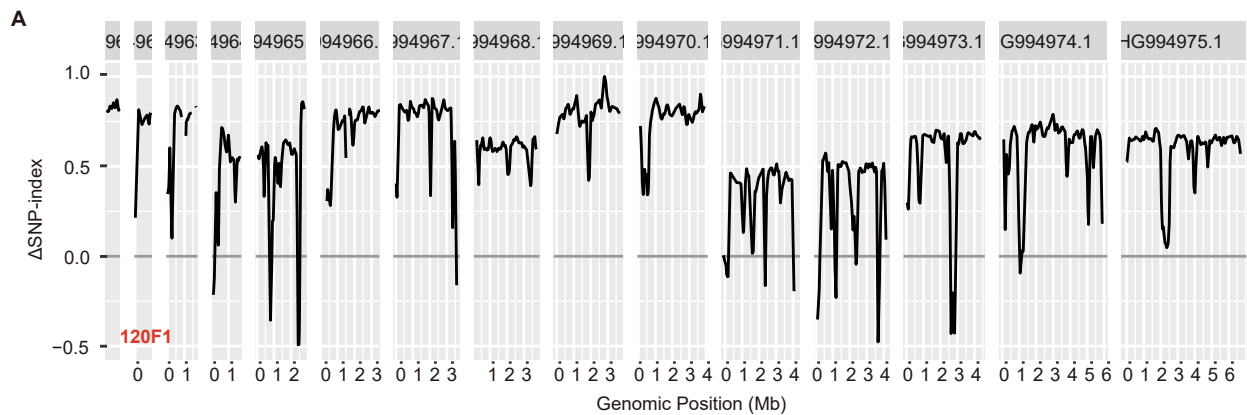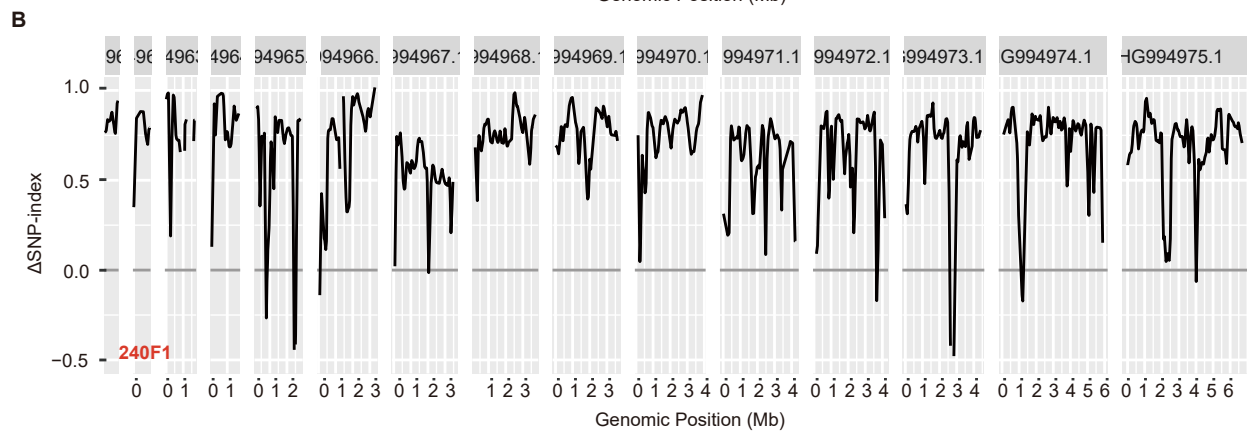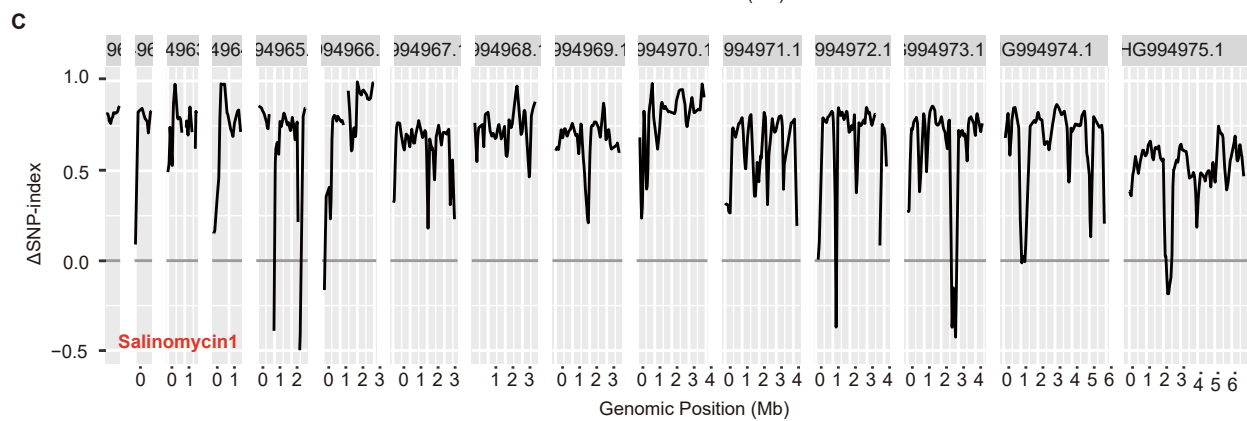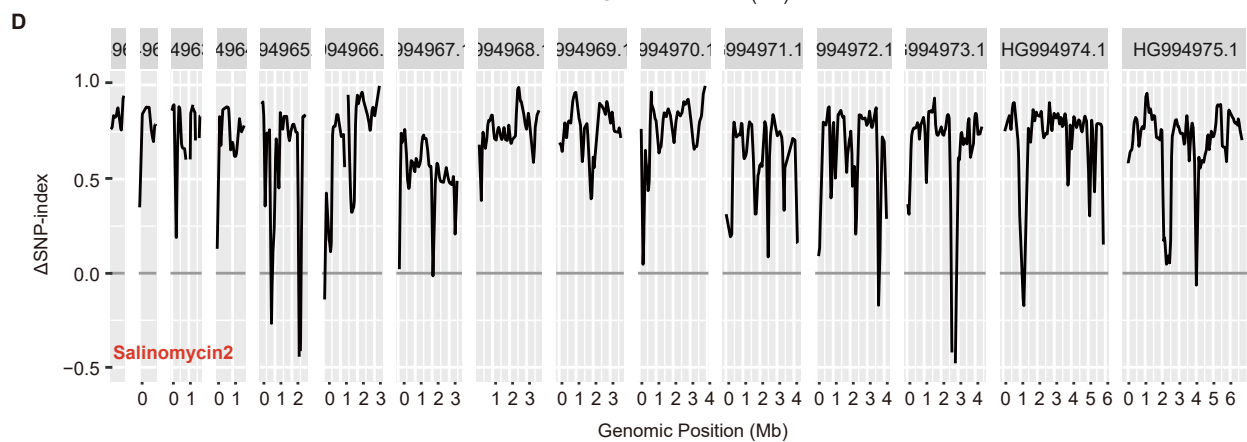

Supplement: Supplementary file 3 — Additional file 3. ΔSNP index analysis of intermediate generations of parasites in the process of experimental evolution. (A-E) QTL analysis of intermediate-generation parasites induced by salinomycin. Quantitative trait loci (QTLs) for salinomycin resistance identified via QTLseq. Plots produced by the plotQTLStats() function with a 20-kb sliding window. [file 13567_2024_1431_MOESM3_ESM.pdf]

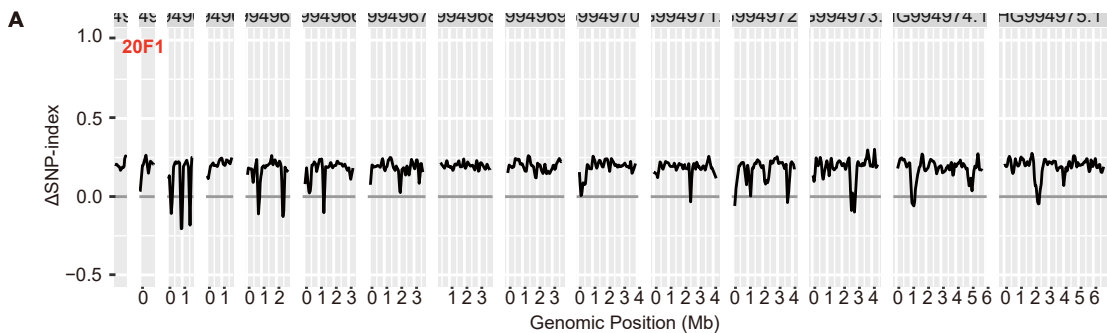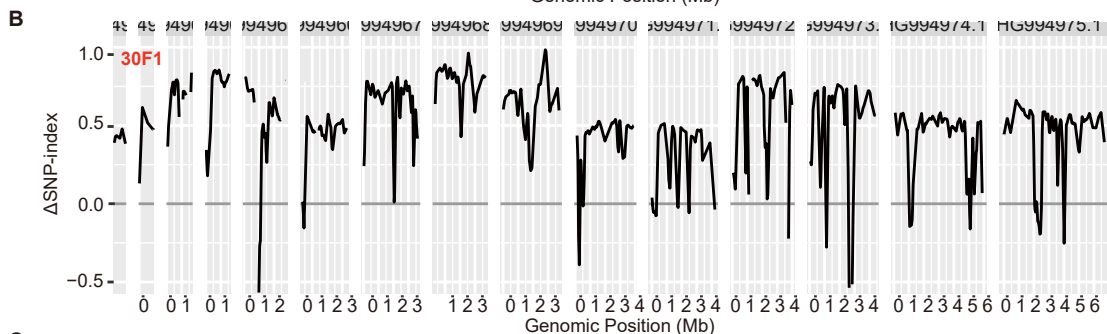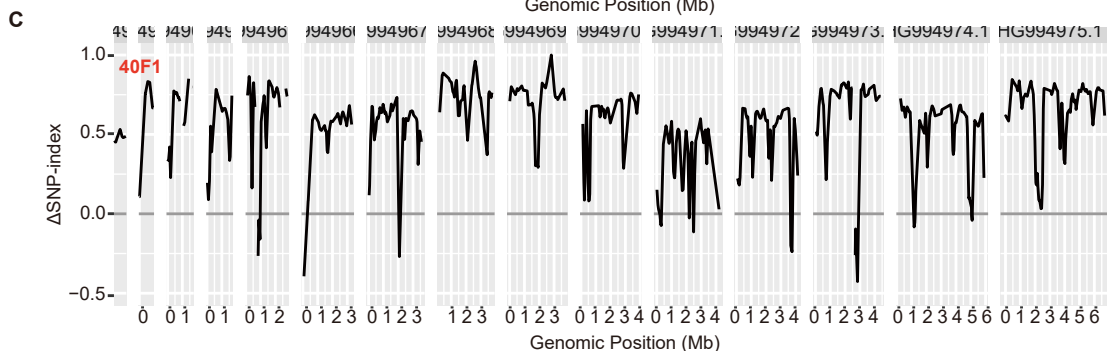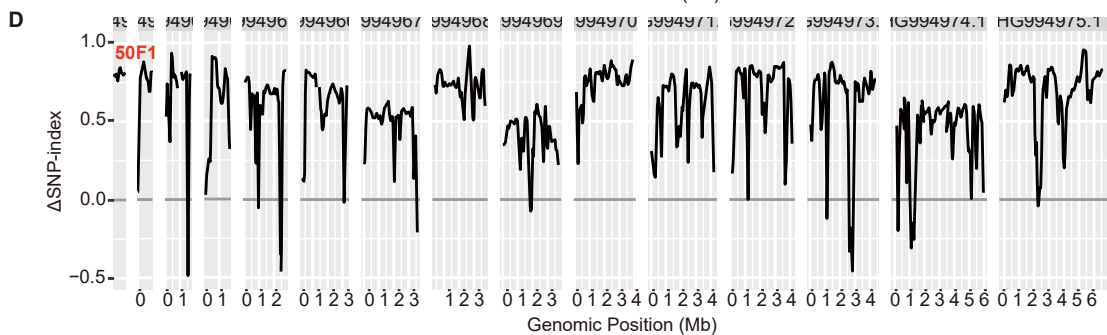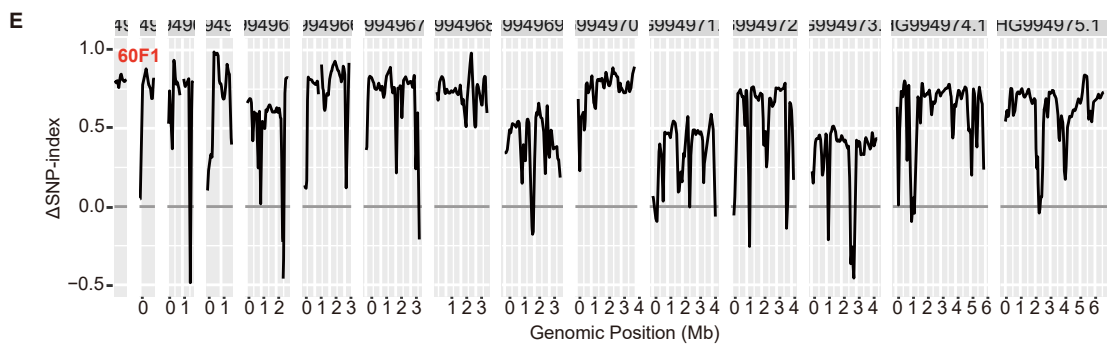

Supplement: Supplementary file 4 — Additional file 4. ΔSNP index analysis of Salinomycin-resistant strains obtained through experimental evolution. (A-D) QTL analysis of strains resistant to salinomycin. Plots produced by the plotQTLStats() function with a 20-kb sliding window. [file 13567_2024_1431_MOESM4_ESM.pdf]
