# Supplementary material for: The F204S mutation in adrenodoxin oxidoreductase drives salinomycin resistance in Eimeria tenella
Source: Vet Res. 2024 Dec 18;55:170. doi: 10.1186/s13567-024-01431-6 (PMC11654014; doi:10.1186/s13567-024-01431-6)
Supplement: Supplementary file 5 — Additional file 5. Acquisition of positive transgenic strains under drug and fluorescence selection. To determine the percentage of positive transgenic parasites, positive sporocysts were collected through flow cytometry and then inoculated with new chickens under salinomycin (240 mg/kg) selection. [file 13567_2024_1431_MOESM5_ESM.docx]

**Additional file 5. Acquisition of positive transgenic strains under drug and fluorescence selection**

|  | **Sample** | **No. of birds/survival** | **Dose of inoculation** | **Day (d)** | **Drug concentration** | **Oocysts output** | **Fluorescent rate** |
| --- | --- | --- | --- | --- | --- | --- | --- |
| 1^st^ | Mutant pool-OE | 15/15 | 1 × 10^7^ | 20 | 240 mg/kg | 3.52 × 10^7^ | 0.3% |
|  | Mutant pool-OE | 5/0 | 1 × 10^7^ | 20 | - | 0 | - |
|  | WT pool-OE strain | 5/5 | 1 × 10^7^ | 20 | 240 mg/kg | 0 | - |
|  | WT pool-OE strain | 5/0 | 1 × 10^7^ | 20 | - | 0 | - |
|  | WT pool-OE strain | 5/3 | 1 × 10^6^ | 20 | - | 6.41 × 10^7^ | - |
|  | WT | 5/5 | 1 × 10^4^ |  | 240 mg/kg | 0 | - |
|  | WT | 5/5 | 1 × 10^4^ | 20 | - | 1.32 × 10^8^ | - |
| 2^nd^ | Mutant pool-OE | 3/3 | 300 | 7 | 120 mg/kg | 9.73 × 10^6^ | 4.1% |
|  | Mutant pool-OE | 3/3 | 500 | 6 | - | 2.6 × 10^7^ | - |
