# Supplementary material for: The F204S mutation in adrenodoxin oxidoreductase drives salinomycin resistance in Eimeria tenella
Source: Vet Res. 2024 Dec 18;55:170. doi: 10.1186/s13567-024-01431-6 (PMC11654014; doi:10.1186/s13567-024-01431-6)
Supplement: Supplementary file 6 — Additional file 6. 3D structure evaluation and basic character prediction. (A) THMHH results for the EtADR protein. Analysis was conducted using the entire amino acid sequence of the EtADR gene. Plots for TMHMM are presented as the probability (y-axis) of an amino acid (x-axis) residue sitting in the helix, inside, or outside summed over all possible model paths. (B) Hydrophobicity plot of the ADR protein. The hydrophobicity values were determined via the method of Kyte and Doolittle. (C) Ramachandran plot analysis of the EtADR 3D protein. The plot calculations were computed via the PROCHECK server. The red regions in the graph indicate the most allowed regions [A, B, L], additional allowed regions [a, b, l, p] are indicated in brown, and generously allowed regions [~a, ~b, ~l, ~p] are indicated in yellow. (D) Details of the residue number in each region of the Ramachandran plot and the G‐score. [file 13567_2024_1431_MOESM6_ESM.pdf]

A

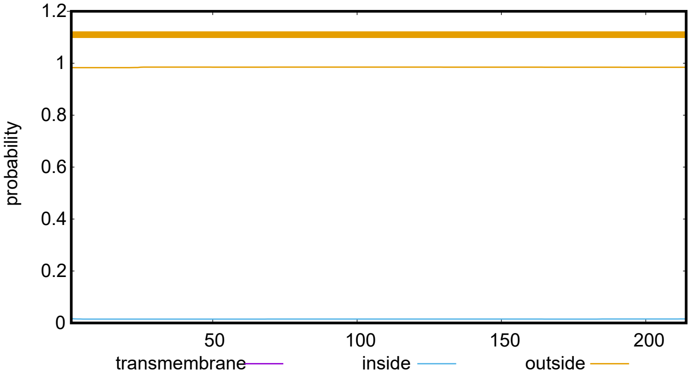

B

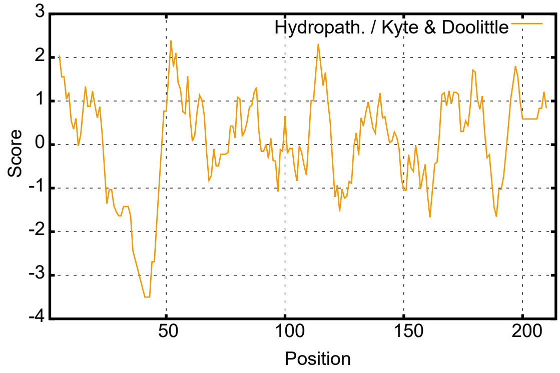

C

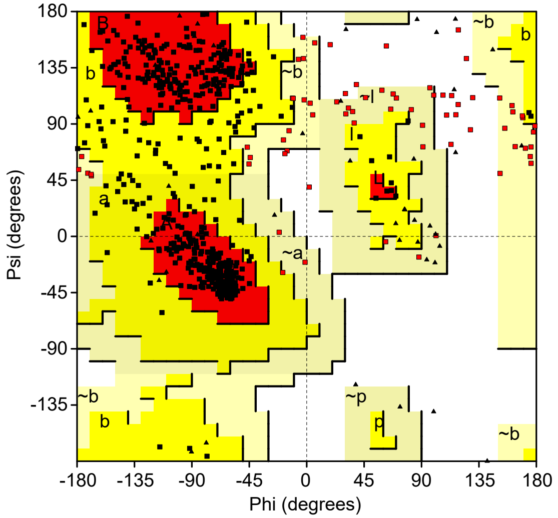

D

	No. of residues	% tage
Residues in most favoured regions [A,B,L]	487	75.3%
Residues in additional allowed regions [a,b,l,p]	91	14.1%
Residues in generously allowed regions [~a,~b,~l,~p]	52	8.0%
Residues in disallowed regions	17	2.6%
	----	------
Number of non-glycine and non-proline residues	647	100.0%
Number of end-residues (excl. Gly and Pro)	1	
Number of glycine residues (shown as triangles)	47	
Number of proline residues	53	

Total number of residues	748	
